# Smart Spotting of Pulmonary TB Cavities Using CT Images

**DOI:** 10.1155/2013/864854

**Published:** 2013-12-03

**Authors:** V. Ezhil Swanly, L. Selvam, P. Mohan Kumar, J. Arokia Renjith, M. Arunachalam, K. L. Shunmuganathan

**Affiliations:** ^1^Computer Science and Engineering, Jeppiaar Engineering College, Rajiv Gandhi Salai, Chennai 119, India; ^2^SVC Polytechnic College, Puliangudi, Tirunelveli DT, Tamilnadu 627855, India; ^3^Department of Computer Science and Engineering, Jeppiaar Engineering College, Chennai, Tamilnadu 600119, India; ^4^KLN College of Information Technology, Pottapalayam, Sivagangai DT, Tamilnadu 630611, India; ^5^Computer Science and Engineering, R.M.K Engineering College, Kavarapettai, Chennai 601206, India

## Abstract

One third of the world's population is thought to have been infected with mycobacterium tuberculosis (TB) with new infection occurring at a rate of about one per second. TB typically attacks the lungs. Indication of cavities in upper lobes of lungs shows the high infection. Traditionally, it has been detected manually by physicians. But the automatic technique proposed in this paper focuses on accurate detection of disease by computed tomography (CT) using computer-aided detection (CAD) system. The various steps of the detection process include the following: (i) image preprocessing, which is done by techniques such as resizing, masking, and Gaussian smoothening, (ii) image egmentation that is implemented by using mean-shift model and gradient vector flow (GVF) model, (iii) feature extraction that can be achieved by Gradient inverse coefficient of variation and circularity measure, and (iv) classification using Bayesian classifier. Experimental results show that its perfection of detecting cavities is very accurate in low false positive rate (FPR).

## 1. Introduction

Even though many effective methods have been taken to reduce the effect of TB, it is a third high rated disease causing death every year since just X-rays are used for detection process. TB cavities near clavicles will not be visible in X-rays. So, dosage estimation for patients would probably go wrong, which results in drug resistance problem. To overcome this problem, an automated segmentation technique is proposed in this work by using CT images. Gradient inverse coefficient of variation and circularity measures is used to classify detected features and confirm true TB cavities.

Classification of the infectious stages based on their intensity is very important to recover from the disease based on the stage of its infection level [1]. Chest radiograph is the primary detection tool [2]. TB diagnosis usually occurs after a combination of skin, blood, and imaging tests. In routine diagnosis, skin and blood tests are taken in case of latent stage of disease in which patients does not show symptoms. CXR is usually taken when the patient shows pulmonary complications. The combination of radiographic findings and demographic/clinical data helps physicians to decide the possibility of infectious TB [3].

Manually the detection of TB cavities is done by just looking at the X-rays/CT images by the doctors/technicians. So by means of looking at the images by the naked eye there is more chance for wrong prediction of the intensity of the cavities. Hence, because of this wrong prediction of the cavities, the physicians may not prescribe correct dosage of medicine. They may prescribe high or low dosage of medicine. If the dosage is too high it will lead to various harmful effects such as causing other diseases. If the dosage is too low the patient cannot easily recover from the disease soon. So the accurate detection of the cavities must be done for the accurate prescription of medicine with the correct dosage to get rid of the disease completely.

Therefore this technique performs accurate detection of cavities, and hence it is used by the physicians to prescribe exact medicine with correct dosage; therefore the patient can recover soon from the disease completely. Tuberculin Skin Testing (TST) or Mantoux test is the standard method of determining whether a person is infected with M. Tuberculosis. Reliable administration and reading of the TST requires standardisation of procedure training, supervision, and practice. Some persons may react to the TST even though they are not infected with TB. But, in some cases, persons may not react to the TST even though they are infected with TB. This makes the detection process complex. By means of the proposed technique, the complexity can be reduced for finding their intensities, and the disease can be identified directly and accurately.

This paper focuses on accurately detecting TB cavities from CT images by CAD system. Currently, the detection of TB cavities from CT images is mainly conducted visually by radiologists based on their knowledge and experience. Computer-aided detection (CADe) and computer-aided diagnosis (CADx) are procedures in medicine that assist doctors in the interpretation of medical images. Imaging techniques in X-ray, Magnetic Resonance Imaging (MRI), and Ultrasound diagnostics yield a great deal of computed tomography. CAD is a technology combining elements of artificial intelligence and digital image processing with radiological image processing. Typical application is in the detection of tumour.

CAD systems are routinely used in a number of medical institutions around the world. It assists radiologists in detection of abnormalities. All commercial computer-aided detection systems use specific threshold values to determine whether an identified suspicious region is ultimately listed as positive findings, and the performance of these systems are frequently evaluated on the basis of the case-based sensitivity achieved at a given false-positive detection rate. It is developed for diagnosis of various diseases and has become commonly used in routine diagnostic procedures such as, the diagnosis of breast cancer and lung cancer [4].

Although numerous techniques for segmenting lung fields have been proposed in [5, 6], automatic detection of TB cavities from CXRs has not been adequately studied. Previous researchers have worked on distinguishing abnormal CXRs from normal ones using texture features as in [7, 8]; however, their approaches do not aim to locate cavities. In this paper, we propose a technique to automatically segment and classify TB cavities from digital CTs. Our method can be applied either independently or as an additional step to previous methods to provide physicians with more information about suspected TB.

Traditionally, X-rays were used for detection process. An X-ray will only produce a two dimensional shadow of an object. It does not provide any details. X-ray beams pass through almost any soft tissue in the body, thereby creating a shadow-like impression of the bones on a radiograph. A shadow will deliver an incomplete picture of an object's shape, thus avoiding the details. Since X-rays creates only a shadow, it is possible that the smaller bones will get overshadowed by the larger shadows. As a result, the cavities near clavicles will not be visible in X-rays. Hence, it is a tough task for the physicians to prescribe medicines. So, CT scan is used in order to generate a three-dimensional image from two-dimensional X-ray picture, a narrow slice at a time. A CT scan thus takes these axial image, compiles them together, and recreates how the tissues and organs are located inside the body, aiding doctors to arrive at an accurate diagnosis. By following this technique, intensity of infection would be accurately determined. The patient can be verified for complete cure, and the treatment can be suspended [9].

**Figure 1:**
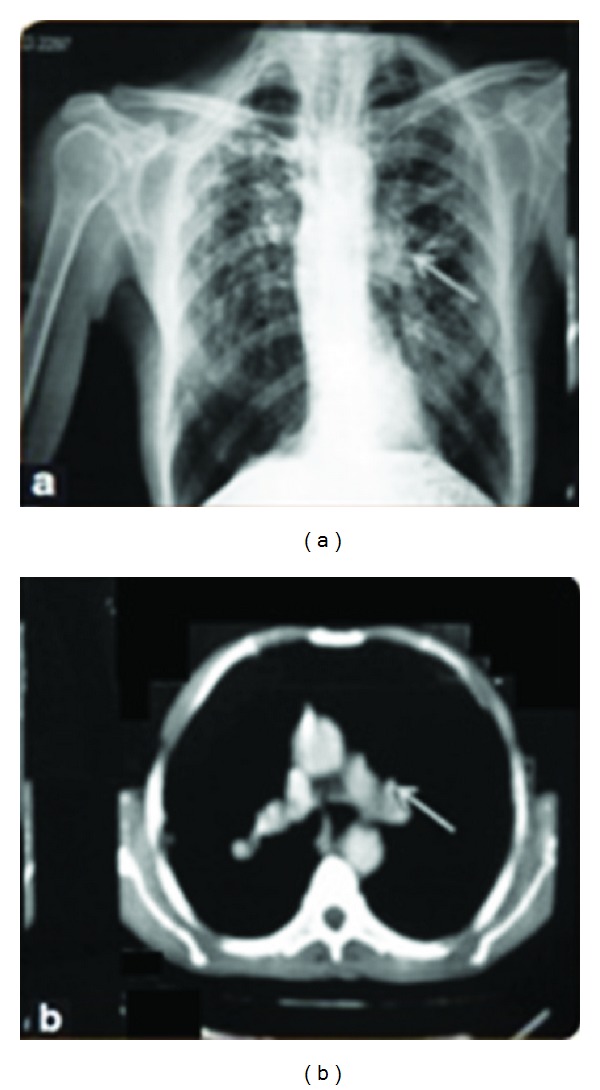
Difference between an X-ray and a CT image, (a) X-ray and (b) CT image.

Figure 1 represents the difference between X-ray and CT images. By analysing the images, we can easily infer that the cavities shown by the arrow in X-ray image are not clear and cannot be viewed properly. But, in case of CT image the arrow clearly indicates the cavities.

The presence of cavities in the upper lobes of the lungs signifies high infection of the disease. Segmentation is an important procedure in medical image analysis and classification especially for radiological evaluation or computer-aided diagnosis. Image segmentation refers to the process of partitioning an image into distinct regions by grouping together neighbourhood pixels based on some predetermined specific properties or features of pixels representing objects in the image. In other words, segmentation is a pixel classification technique that allows the formation of regions of similarities in the image [10]. Image segmentation methods can be broadly classified into three categories: boundary-based techniques, region-based techniques, and pixel-based direct classification methods. In practice, region-based methods are mostly used. They are typically very fast and easy to manipulate [11].

To improve performance of region-based methods and their results, preprocessing techniques are usually required. The segmentation of the cavities should be done automatically for which active contour (AC) models (or snakes) [12–14] are commonly used as segmentation techniques for medical images. ACs are computer generated curves that move within image to find object boundaries. They can be used to track objects in temporal as well as spatial dimensions. They can easily be manipulated using external GVF forces. The GVF forces are used to derive the snake, modeled as a physical object having a resistance to both stretching and bending, towards the boundaries of the object.

The GVF forces are calculated by applying diffusion equations to both components of the gradient of an image edge map. Active contour is done after the initial contour for locating a particular intensity of the cavity. In this initial contour method, Mean Shift Segmentation Algorithm is used. AC models have limited capture range. It cannot converge accurately unless an initial contour is specified close to the region of interest. Since more than one TB cavity is possible in infected lungs, we assess the performance of AC models in segmenting multiple cavities. A common weakness of most AC models is the necessity to define an initial contour close to the Region of Interest (ROI) for the algorithms to evolve and converge.

Various Preprocessing techniques have been used to remove normal or unrelated structures in the CT images. However, analysis of the CTs shows that the conditions, such as the texture and shape, in the individual lungs could vary significantly. Therefore, subtraction techniques cannot be directly applied. To deal with the complicated texture and varied intensity distribution, a clustering and adaptive thresholding scheme coupled with a robust AC model is applied in our approach to extract suspected cavities. True cavities are then distinguished from false positives (FPs), using a Bayesian classifier. Both the segmentation and classification are automatic. We believe that our work is one of the first attempts at automatically locating pulmonary TB cavities in CTs with high-accuracy and low FPR. In [15], the authors have proposed a technique to detect TB in microscopic images. They have used adaptive threshold based segmentation and morphological, colour, and size filters to remove unwanted data set in the (R-G) Segmented image.

In [16], a technique to detect TB has been proposed, based on binarization process to set edge lines of ribs, Gradient vector flow model for segmentation and K-means algorithm for classification. Also, in [17], a method has been discussed to detect TB Ziehl-Neelsen Stained Tissue Slide Images. Active shape model was used for texture analysis. Multilevel Image Enhancement for Pulmonary TB Analysis is the task of autodetecting the tiny nodules, which will help to get more information of pulmonary tuberculosis. Two image processing techniques were applied into lung tissue information recognition. (1) A repetitive smoothing-sharpening technique is proposed and its impact is assessed to beneficially enhance X-ray lung images. (2) The ridge detection algorithm is going to diagnose indeterminate nodules correctly, allowing curative resection of early-stage malignant nodules and avoiding the morbidity and mortality of surgery for benign nodules. The proposed technique has been tested on lung X-ray images [18].

In [19], the authors have proposed an efficient coarse-to-fine dual scale technique for cavity detection in chest radiographs. Gaussian-based matching, local binary pattern, and gradient orientation features are applied at the coarse scale, while circularity, gradient inverse coefficient of variation and Kullback-Leibler divergence measures are applied at the fine scale. A survey of Automatic screening for TB in chest radiographs shows that TB screening is a challenging task and an open research problem [20].

## 2. Preprocessing and Experimental Setup

The materials used in this paper are the CT images. They are pre-processed before segmentation and classification process. Image preprocessing can significantly increase the reliability of an optical inspection. Preprocessing is very useful in a variety of situations since it helps to suppress information that is not relevant to the specific image processing or analysis task. Therefore, the aim of preprocessing is an improvement of the image data that suppresses undesired distortions or enhances some image features important for further processing. Although geometric transformations of images (e.g., rotation, scaling, and translation) are also classified as preprocessing methods, in this model, we have used techniques such as image resizing, masking, and Gaussian smoothening.

**Figure 2:**
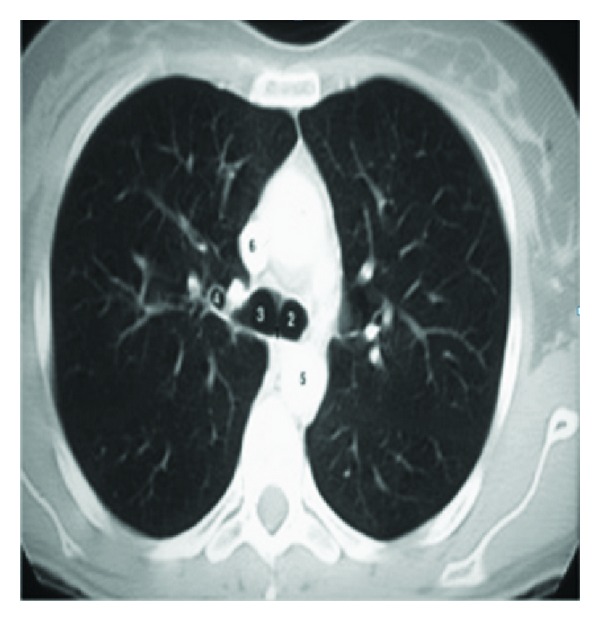
Original CXRs with TB cavities often have low-contrast and complicated texture.

**Figure 3:**
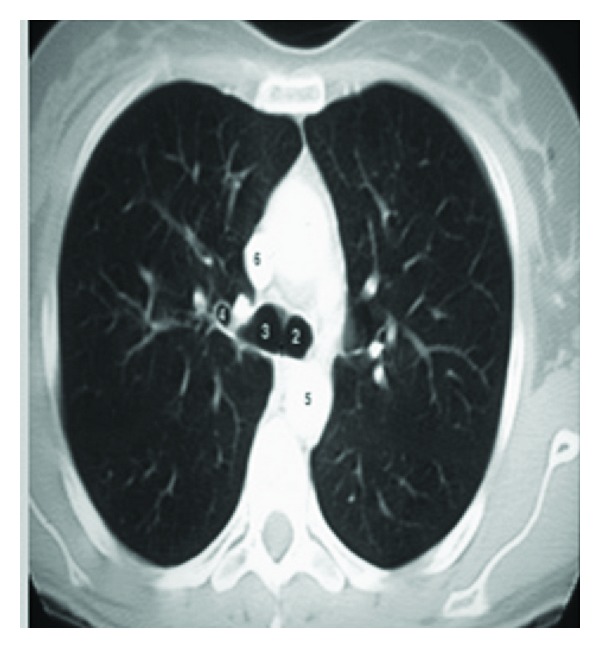
Resized image.

In image resizing, the size of an image is increased. So, the pixels which comprise the image become increasingly visible, making the image soft as shown in Figure 3. Image masking has to be done to remove background (outer area of a lung) area of an image. Gaussian smoothening is done to enhance the contrast while reducing the noise caused by the complicated texture. These images usually have low contrast and complicated texture. The complex patterns surrounding a TB cavity are indicated by numbers as in Figure 2. Therefore, it is difficult to directly apply these methods without adaptation for lungs infected with TB. Instead, after examining 30 pre-processed images, a mask is created to approximate the Upper Lower Zones (ULZs), as shown in Figure 5.

**Figure 4:**
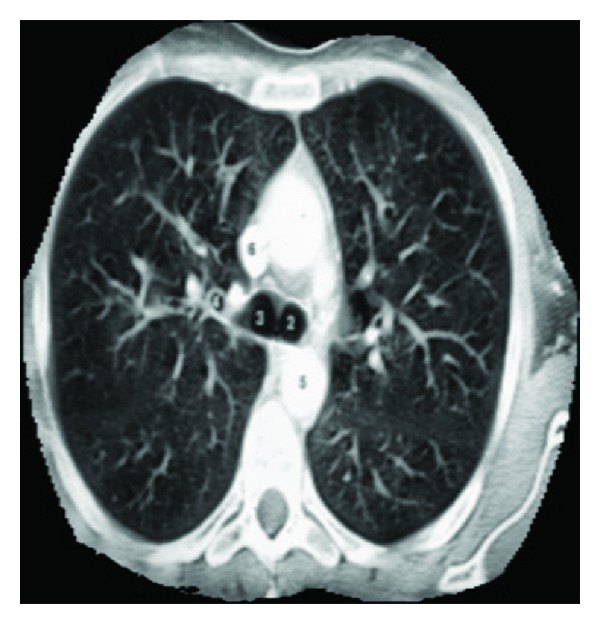
Image after Preprocessing of original image.

After preprocessing, the preprocessed image as in Figure 4 is obtained, for which the segmentation is carried out. It is done by two methods, initial contour and the active contour. In the initial contour method, the technique known as mean shift segmentation model is used. Mean shift segmentation is classified as density estimation function. In the active contour gradient vector flow technique is used. The GVF forces are used to derive the snake, modeled as a physical object having a resistance to both stretching and bending, towards the boundaries of the object. The GVF forces are calculated by applying diffusion equations to both components of the gradient of an image edge map. They can be used to track objects in temporal as well as the spatial dimensions. They can easily be manipulated using external GVF forces.

## 3. Cavities Classification Model

From many other segmentation methods proposed in the literature, the initialization step in our solution is fully automatic, which is necessary when a large number of CTs need to be examined. Initialization is carried out in three stages. In the first stage, it deals with the preprocessing of images. In the second stage, it deals with segmentation in which setting threshold and clustering of the pixel are carried out to define initial contours of suspected cavities [21]. In the initial contour method the technique known as mean shift segmentation model is used. Mean shift segmentation is classified as density estimation function. The initial contours are then converged using an AC model by means of GVF segmentation algorithm.

**Figure 5:**
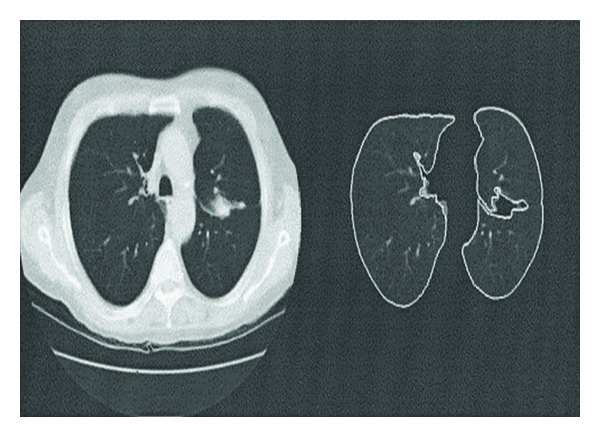
Image masking.

In the third stage, the suspected TB cavities are either confirmed or excluded by using optimal thresholds in a Bayesian classification technique based on gradient inverse coefficient of variation (GICOV) [22] and mean circularity measure [23]. If a cavity appears near the clavicles, it is very likely that the cavity is partially occluded, which makes the visible part of the cavity violate the circularity criterion. In addition, as compared in Figure 5, the radio-dense clavicles make the intensity distribution of lung fields near them quite different from other portions of the lung fields which may require a different GICOV threshold. Therefore, if no cavity is confirmed in the clavicle regions in second stage, those regions go through segmentation and classification stage (third stage), using new GICOV and circularity thresholds calculated for them.

**Figure 6:**
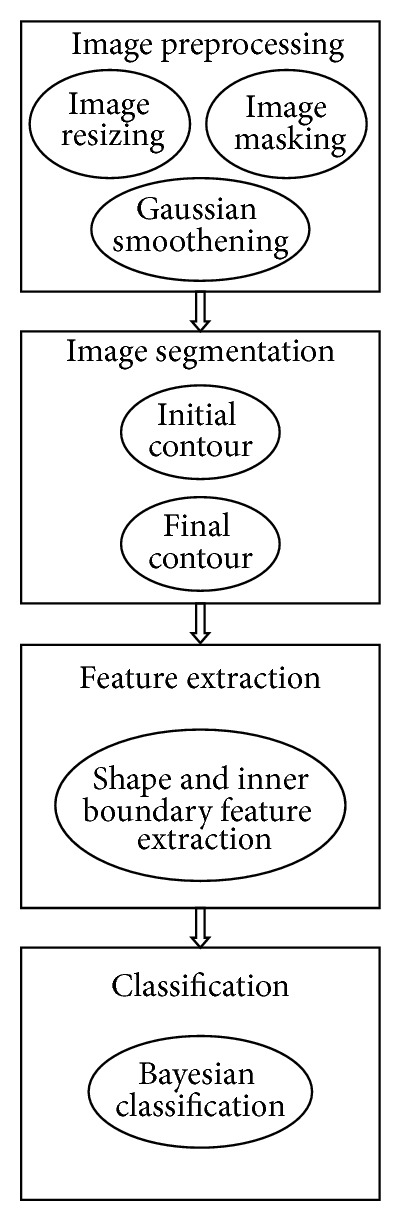
Architecture of cavities classification model.

**Figure 7:**
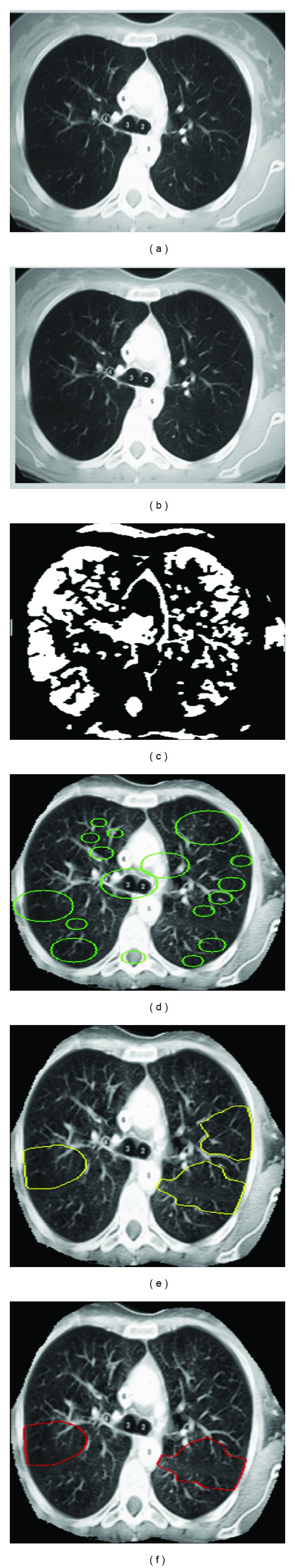
Sample images obtained during the classification process steps: (a) pre-processed, (b) mean shift-segmented, (c) thresholded, (d) initial contours, (e) active contour segmented, and (f) cavities classified.

An overview of the cavities classifying model is given in Figure 6. In Figures 7(a)–7(f), the sample images obtained during the classification process steps have been shown.

### 3.1. Image Preprocessing

Removing unwanted dataset in the image is done by using the following preprocessing techniques: image resizing, image masking, Gaussian smoothening, and conversion of unsigned images into double precision images. Image resizing is by minimizing an image into 300 × 200, since normal CT image of a lung would be more than 1000 × 500. Masking is done in such a way that there are two bitmaps in the actual image. The unused areas are given a pixel value with all bits set to 0s. In a masked image, the image areas are given a pixel value of all bits set to 0s and the surrounding areas a value of all bits set to 1s.

Histogram equalization is a method in image processing for contrast adjustment. Using the image's histogram through this adjustment, the intensities can be better distributed on the histogram. This allows for areas of lower local contrast to gain a higher contrast. Histogram equalization accomplishes this by spreading out the most frequent intensity values such that the data set will be clearer. As Gaussian noise is used, at each pixel, the values are statistically independent.

### 3.2. Image Segmentation

Segmentation refers to the process of partitioning an image into multiple segments (set of pixels, called super pixels). Image segmentation is used to locate objects and boundaries (lines, curves) in images. It is the process of assigning a label to every pixel in an image, so that pixels with the same label can have some similar characteristics. For the mean shift segmentation of initial contour, feature space parameter will be considered as probability density function (probability of likelihood of a variable to occur in a certain region will be integrated together and results will be calculated).

Although AC models based on energy minimization are effective segmentation methods, the traditional AC models have several drawbacks: they are sensitive to the initial contour placement, can have a capture range too small to detect cavities, may need to apply different parameter values to guide convergence, and do not perform well on weak edges [24]. To deal with these problems and take advantage of the knowledge that the boundaries of TB cavities follow dark-to-bright transitions in CTs, we incorporate the directional GVF models to delineate the suspected TB cavities. This AC model performs well for contrast changes and weak edges, since multiple cavities can exist as in Figure 2. In contrast to other AC models, which require an initial contour to be selected manually, we automate the initial contour placement using mean shift segmentation as in Figure 7(b) and apply adaptive thresholding to control the clustering process. Mean shift [25] is a feature space analysis technique that clusters neighbouring data points with similar characteristics using a neighbourhood search procedure.

Selecting a global threshold for the entire image cannot produce accurate result because of the varied intensity distribution. In our adaptive threshold approach, the threshold needs to be determined by local image attributes. This will reflect not only the characteristics in the neighbourhood but also the presence of TB cavities. In order to discover such an attribute, we perform experiments using the training image set. Due to the destruction of pulmonary tissue caused by TB, the texture of one lung is very likely to be quite different from the other in the same CT [26]. Therefore, different thresholds should be applied to individual lungs according to their contents.

The existing system for the detection of cavities has been done by using the CXR, where the cavities near the clavicles are tough to identify. But in the proposed system, we focus on CT images as CT images give three dimensional view and accurate picture of the cavities near clavicles that can be viewed clearly, and hence detection is done accurately. The proposed system uses Mean Shift and GVF segmentation algorithms for image segmentation.

#### 3.2.1. Mean Shift Segmentation Algorithm

The mean shift technique detects modes in a probability density function based on the Parzen Density Estimate as follows [26]:

(1) f^ks(x)=1Nhd∑i=1NKs(x−xih),

where *N* is the number of *d*-dimensional vectors *x*_1_,…, *x*_*N*_. The parameter *h* is the window radius of the kernel *K*_*s*_. During image segmentation, each feature vector contains spatial information of each pixel and the corresponding colour/intensity information in the range domain of dimension one or more.

The multivariate mean shift vector in any point *p* can be calculated as

(2) $$\begin{array}{c} m_{k}\left(p\right)=\frac{\sum_{i=1}^{N}p_{i}K\left((p-p_{i})/h\right)}{\sum_{i=1}^{N}K\left((p-p_{i})/h\right)}-P. \end{array}$$

So, considering a uniform kernel, the mean shift vector can be calculated from the above equation gets the value of the average calculated from the differences in vectors. So, it is evident that the mean shift vector is proportional to the normalized density gradient estimate [27] as follows:

(3) mk(p)=12h2c∇f^KE(p)f^KU(p),

where *K*_*E*_ can be calculated as

(4) $$\begin{array}{c} K_{E}\left(X\right)=\{\begin{array}{cc} \frac{1}{2}C_{d}^{-1}\left(d+2\right)\left(1-{||p||}^{2}\right) & ||p||\leq 1\\ 0 & \text{Otherwise.} \end{array} \end{array}$$

The algorithm for Mean shift segmentation is as follows.

 
*Input.* Pre-processed Image
 
*Output.* Suspected area in the image (circled parts in the image)
 
*Step 1.* Set up 100 × 100 pixel as a seed point (mean value)
 
*Step 2.* Initialize the threshold value
 
*Step 3.* Calculate the distance from the mean to each pixel in the image
 
*Step 4.* Square the distance value and check if it is lesser than the threshold; then add those pixels with the cluster
 
*Step 5.* Calculate new mean in the cluster
 
*Step 6.* If both the clusters (pixels in clusters) are lesser than the threshold/2
   Group them as one
 Else
   Redo the steps from Step 3 based on the new mean
 
*Step 7*. Get all the connected components (patches) in each cluster
 
*Step 8*. Compare each patch in the cluster with neighbourhood clusters which adds votes in an array for each pixel
 
*Step 9*. Finally group the pixels which are all having more votes for a cluster.

We have taken 5 as threshold value for the purpose of clustering; that is, five times the pixel value is compared with the neighbourhood pixels and then grouped together into a single cluster. And hence clustering is done by using threshold value. The seed (mean) point is picked randomly. This seed point is used as a start of mean. Mean points are used for pointing the location. The seed point can be 120 × 100, 150 × 200, and so on. During experimentation it was found that the segmentation process showed improved performance when the seed point was chosen as 100 × 100.

#### 3.2.2. GVF Segmentation Algorithm

The Gradient Vector Flow (GVF) framework defines a new well designed, bi-directional external force that identifies the object boundaries from both of the directions and can deal with concave regions. GVF is an alternative to the distance transform. Unlike distance transform, a binary edge map is required; the GVF is estimated directly from the continuous gradient space. Also, the diffusion process that provides the GVF leads to a measurement that is contextual and not equivalent to the distance from the closest point. This is because more than one boundary pixel take part in the estimation of the GVF.

A Gaussian edge detector is used to define a continuous edge based data space. It is also assumed that it has mean value as zero and variance as *σ*_*E*_ as follows:

(5) $$\begin{array}{c} g\left(p\right)=\frac{1}{2\pi \sqrt{\sigma_{E}}}e^{-{\left|\nabla (G_{\sigma }*I)(p)\right|}^{2}/{2\sigma }_{E}^{2}},\;\;f\left(x,y\right)=1-g\left(p\right). \end{array}$$

The GVF segmentation algorithm is as follows.

 
*Input.* Output of Initial Contour
 
*Output.* Deformed shape of circles
 
*Step 1.* To deform the shape (change circle shape to compressed or stretched form) solve a linear equation as follows:
   
*ax* = *b*
   
*a*→ matrix (*o*/*p* from initial contour)
   
*b*→ vector field
   Calculate the GVF force
 
*Step 2*. Interpolate the deformed shape using GVF force calculated above
   If shape compressed too much
   Remove a neighbourhood pixel
 Else
   Add some pixels in between randomly
 
*Step 3.* Repeat Step 2 for more iteration and stop if there is no change in shape
 
*Step 4.* Give some colour to differentiate the circle from the Image.

### 3.3. Image Classification

After the TB cavities are segmented out from the images, they are classified using a Bayesian classifier. Two features are considered for classification: the inner boundary of a TB cavity which has dark-bright transition and the TB cavity which often appears circular in CT. To best represent the two features, we use GICOV algorithm for inner boundary features and circular measure for shape feature [27].

In the Bayesian analysis, prior probabilities are based on previous experience. In this case cavity and noncavity images are considered for prior probability.

Thus, we can write the following:

(6) Prior  probability  for  Cavity  Image  ∝Number  of  Images  with  cavitiesTotal  Number  of  images,Prior  probability  for  Noncavity  Image  ∝Number  of  Noncavity  ImagesTotal  Number  of  images.

This classification step is used to detect cavities in region near clavicles. If we fail to detect the cavities in the previous two steps and the cavities detected in this area do not satisfy the circular condition, the higher threshold value is used for classification. This model provides the detection framework that uses the classification measures such as GICOV, circularity, and the hybrid approach in identifying TB cavities.

#### 3.3.1. Gradient Inverse Coefficient of Variation Algorithm

Let (*X*(*s*), *Y*(*s*)) represent a 2-D closed contour parameterized through *s* ∈ [0, 1]. If *I*(*x*, *y*) denotes an image, then the mean of the image gradient *M*(*X*, *Y*) over the entire contour computed in the outward normal direction is given by

(7) $$\begin{array}{c} M\left(X,Y\right)=\frac{1}{L}\int_{0}^{1}\nabla I\left(X\left(s\right),Y\left(s\right)\right)\cdot n\left(X\left(s\right),Y\left(s\right)\right)ds, \end{array}$$

where *n*(*X*(*s*), *Y*(*s*)) is the unit outward normal to the contour at (*X*(*s*), *Y*(*s*)) and *L* is the length of the contour given by

(8) $$\begin{array}{c} L=\int_{0}^{1}\sqrt{{X_{S}}^{2}+{Y_{S}}^{2}}ds. \end{array}$$

The incorporation of directional information provides superior results when the contour intersects adjacent object boundaries. When the snake model is implemented without respect to directionality, the snake may be attracted to a strong edge that has a different gradient direction and could be resultant from another object. The use of the gradient operator as a segmentation/edge detection tool is substantially enhanced by the utilization of directional information [27].

The variance of the image gradients over the entire contour computed in the outward normal direction n is given by

(9) S2(X,Y)=1L∫01[∇I(X(s),Y(s))·n(X(s),Y(s))]2ds−M2(X,Y).

It is important to note that even if the object edge strength was not quite uniform along its boundary, in a highly cluttered environment the statistic [8] would be quite effective in rejecting clutter. Taking into account the above considerations, we can define the GICOV as

(10) $$\begin{array}{c} V\left(X,Y\right)=\frac{M\left(X,Y\right)}{S\left(X,Y\right)/\sqrt{L}}, \end{array}$$

where $\sqrt{L}$ functions as a normalization factor. The algorithm for GICOV is as follows:

 
*Input.* Threshold image, suspected area (circles)
 
*Output.* Deformed image
 
*Step 1.* Calculate the GICOV value for each point
   Based on the mathematical model proposed by Dong et al.,
     
*n*→ denotes the points on the circle
     
*s*→ standard deviation of points
     
*m*→ constant value
 
*Step 2.* Select the circles based on gamma and *m* values given
 
*Step 3.* Find out the pixels which are local maximum of GICOV values inside each circle
 
*Step 4.* Recalculate the above procedure until the values are constant.

#### 3.3.2. Algorithm for Circularity Measure

A circularity measure can be estimated by using the similarity measure of polygons. The important thing is to find out the similarity between an input discrete curve and a real circle. Initially, the tangent space representation of a real circle has to be determined. The circularity measure can be defined as follows:

(11) $$\begin{array}{c} {\text{CM}}_{\varsigma }=\int_{0}^{1}{\left(T\left(\varsigma \right)\left(s\right)-2\pi s+\theta_{0}\right)}^{2}ds, \end{array}$$

where *θ*_0_ = ∫_0_^1^ *T*(*ς*)(*s*)*ds* − *π*.

The algorithm for estimating the circularity is as follows:

 
*Input.* Circled area
 
*Output.* Circles (having bright to dark transition)
 
*Step 1.* Calculate mean value between points on the curve of the suspected area, which is the output of the gradient inverse coefficient of variation module.
 
*Step 2.* Find out the square root of mean value of points on the curve. Find variance for the value which is calculated above.
 
*Step 3.* Find maximum value of square root, which is calculated above.
 
*Step 4.* Calculate circularity factor by, dividing the variance value by max (square root)
 
*Step 5.* Stop this iteration at the point of getting repeated values. Return the circularity factor to the main program.

#### 3.3.3. Hybrid Scheme

In the hybrid scheme, we have used GICOV algorithm for inner boundary features and circular measure for shape feature together for classification.

## 4. Results and Discussion

The CT images used for testing and training for classification and segmentation were collected from Sree Moohambika Institute Of Medical Science, Kulasekaram, and Trivandram Medical College, India. They were freely collected for research purpose. We have used 54 CT images, out of which 17 images have cavities and the remaining images do not have cavities.

For detecting cavities before handling the classification technique, the segmentation of the image takes place, in which initial contour is done before active contour. For larger number of CTs to be examined, this initial contour method is impossible. In computer aided system, the approach is to locate the cavities in the lung field and start the snake evolution. The automatic step is initialized by choosing mid-point in each of the images which is roughly in the middle of ULZ, to initialize a circle with a radius of three pixels as an initial snake [26]. The set of cavity detected CT images and noncavity CT images is tested to give an accurate result. Thus this technique deals with the accurate detection of cavities.

**Figure 8:**
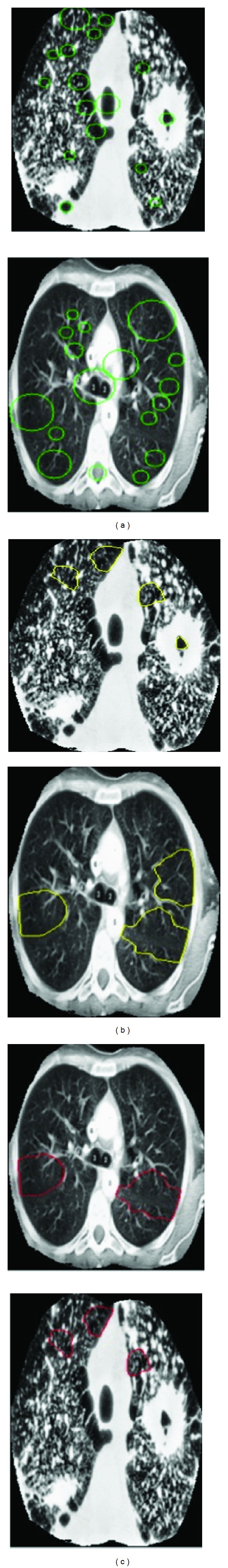
Comparison of performance using different classification measures on two test images. Using the hybrid classification measure produces fewer FPs than using GICOV alone or using circularity alone. (a) Initial contour, (b) active contour, and (c) cavities.

Figure 8 shows the segmentation results using our algorithm on two test images. Cavities, active contour, and initial contour are represented in red, yellow, and green colours, respectively. All the initial contours (green circles) in this comparison are generated automatically, using our method [28].

### 4.1. Performance Evaluation

We have performed statistical analysis by comparing the performance of the proposed system with CXR images and with CT images.  Table 1 compares the performance of the system with CXR images with different features. In Table 2, different features were used for classification, and the images used were CT images. The experiments were conducted with CT images for which results were already known (lungs which were affected with TB and not affected). The normal set contains CT images without TB infection. The noncavity set seems to have cavities during manual inspection, but actually it is a noncavity image. All set contains normal images, cavity images, noncavity images.

The above comparison has been done by examining both CT images and CXR images. First, the processing has been started by providing initial contour on every image. Then the classification has been done based on the GICOV, circularity, and hybrid methods. The hybrid approach shows a best performance among the three with the FPR as 0.143/image. On the other hand, the rate is almost double when using GICOV alone or circularity alone.

**Figure 9:**
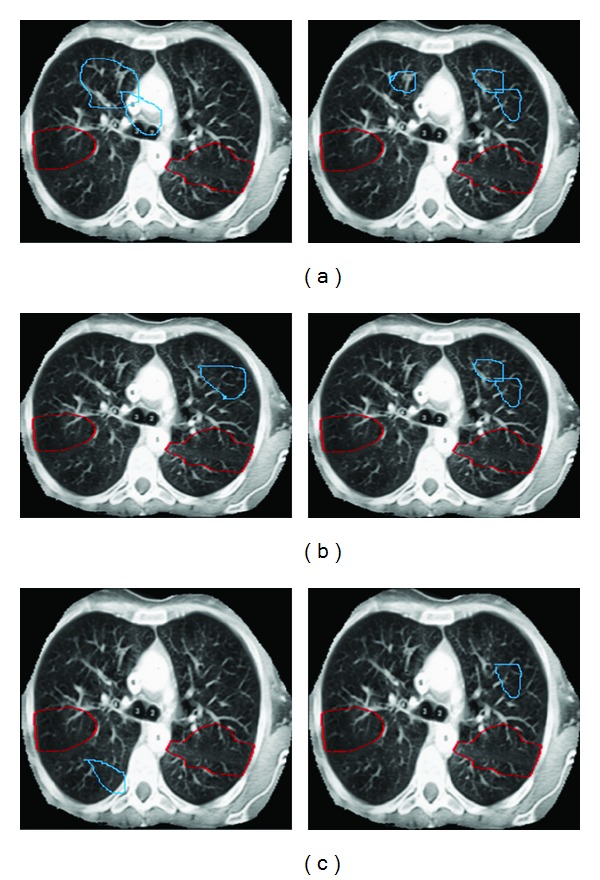
Comparison of performance based on different CT images (a) GICOV (b) circularity (c) hybrid.

The comparison of two CT test images based on different classification is depicted in Figure 9. In the images on left side, GICOV alone produces 2FPs, while the circularity alone and hybrid approach produce only 1FP. The right side image shows that GICOV alone produces 3FPs, the circularity alone produces 2FPs, and hybrid approach produces only 1FP. Thus the performance is good in hybrid approach.

### 4.2. Training and Testing

The digital images to be tested are copied to CAD server in a DICOM-format, and we have prepared and analysed in several steps.

 
*Step 1.* Preprocessing is used for
   (a) reduction of bugs in images
   (b) image noise reduction
   (c) levelling of image quality for clearing the images different basic conditions
 
*Step 2.* Segmentation is used for
   (a) differentiation of different structures in the image, for example, lungs, ribcage, and possible round lesion
 
*Step 3.* Structure/ROI (region of interest) analyse every detected region individually for
   (a) compactness
   (b) form, size, and location
   (c) reference to close-by structures
 
*Step 4.* Feature extraction for
   (a) shape features
   (b) inner boundary features
 
*Step 5.* Bayesian classification for
   (a) classification TP and FP of the cavities.

### 4.3. Limitation of the Approach

**Figure 10:**
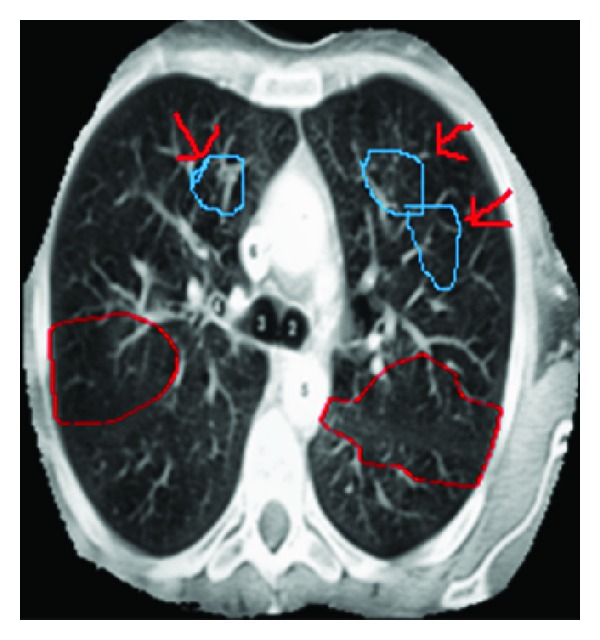
Two cavities are missed by our technique because automatic initialization fails to place initial contours inside the cavities.

In Figure 10, there are cavities missed by our method. This problem arises because automatic initialization fails to place initial contours inside the cavities. To overcome these missing cavities, features such as adaptive thresholding and template-based schemes can be used in the future. By using these template-based schemes, the cavities which have been failed to detect will be also detected.

## 5. Conclusion and Future Work

In this paper, an approach for automatic TB cavity detection from CTs has been proposed. So far, there are no automatic algorithms developed that can detect TB cavities from CTs accurately. In this approach, a mean shift segmentation technique integrated with adaptive thresholding to automate the initial contour has been done. These initial contours are further used in a GVF snake model to segment out suspected features. In the subsequent classification process, GICOV and circularity thresholds are applied. Experimental results demonstrate that our method achieves good accuracy with a low FPR.

## Figures and Tables

**Table 1:** Comparison of performance using X-ray images.

	Cavity set		Noncavity set		Normal set		All	
	TPR	FPR (per image)	TPR	FPR (per image)	TPR	FPR (per image)	TPR	FPR (per image)
GICOV	74.1	0.984	79.4	0.785	70.4	0.028	80.1	0.189
Circularity	75.4	0.915	78.8	0.641	72.9	0.0187	80.9	0.195
Hybrid	76.2	0.685	74.9	0.342	75.8	0.0096	81.2	0.18

**Table 2:** Comparison of performance using CT images.

	Cavity set		Noncavity set		Normal set		All	
	TPR	FPR (per image)	TPR	FPR (per image)	TPR	FPR (per image)	TPR	FPR (per image)
GICOV	80.2	1.043	81.1	0.803	80.5	0.043	82.5	0.243
Circularity	80.5	1.243	80.8	0.59	81.2	0.033	82.3	0.23
Hybrid	81.4	0.743	80.3	0.443	82.5	0.02	83.3	0.143
